# Five-Year Outcomes After Arthroscopic Surgery for Femoroacetabular Impingement Syndrome in Elite Athletes

**DOI:** 10.1177/0363546520908840

**Published:** 2020-03-20

**Authors:** Ida Lindman, Axel Öhlin, Neel Desai, Kristian Samuelsson, Olufemi R. Ayeni, Eric Hamrin Senorski, Mikael Sansone

**Affiliations:** † Department of Orthopaedics, Institute of Clinical Sciences, Sahlgrenska Academy, Gothenburg University, Gothenburg, Sweden; ‡ Division of Orthopaedic Surgery, McMaster University, Hamilton, Canada; § Orthocenter/IFK-Kliniken, Gothenburg, Sweden; Investigation performed at Orthocenter/IFK-Kliniken, Gothenburg, Sweden, and Department of Orthopaedics, Sahlgrenska Academy, Gothenburg, Sweden

**Keywords:** hip, femoroacetabular impingement, hip arthroscopy, elite athletes, outcomes

## Abstract

**Background:**

Femoroacetabular impingement syndrome (FAIS) is a common cause of hip pain and disability in athletes. Arthroscopic treatment for FAIS is well-established; however, the long-term results in elite athletes are limited.

**Purpose:**

To evaluate outcomes 5 years after arthroscopic treatment for FAIS in elite athletes.

**Study Design:**

Case series; Level of evidence, 4.

**Methods:**

Elite athletes undergoing arthroscopic treatment for FAIS with a minimum 5-year follow-up were included. They were prospectively followed up with patient-reported outcome measures. An elite athlete was defined as having a Hip Sports Activity Scale (HSAS) level of 7 or 8 before the onset of symptoms. Preoperatively and 5 years after surgery, all athletes completed a web-based questionnaire, including the Copenhagen Hip and Groin Outcome Score (comprising 6 subscales), the EQ-5D and EQ-VAS (European Quality of Life–5 Dimensions Questionnaire and European Quality of Life–Visual Analog Scale), iHOT-12 (International Hip Outcome Tool), a visual analog scale for hip function, and the HSAS. Moreover, patients reported their overall satisfaction with their hip function. Preoperative measurements were compared with the 5-year follow-up.

**Results:**

A total of 64 elite athletes (52 men, 12 women) with a mean ± SD age of 24 ± 6 years were included. On average, patients reported a statistically significant and clinically relevant improvement from preoperative patient-reported outcome measures to the 5-year follow-up (*P* < .0003), Copenhagen Hip and Groin Outcome Score subscales (symptoms, 51.7 vs 71.9; pain, 61.0 vs 81.1; function of daily living, 67.1 vs 83.6; function in sports and recreation, 40.0 vs 71.5; participation in physical activity, 25.0 vs 67.4; hip and groin–related quality of life, 34.4 vs 68.0), EQ-5D (0.60 vs 0.83), EQ-VAS (66.1 vs 76.7), and iHOT-12 (40.0 vs 68.8). At the 5-year follow-up, 90.5% reported satisfaction with their overall hip function. In total, 54% still participated in competitive sports (HSAS, 5-8) at follow-up, while 77% had decreased their level. Older patients and patients with longer duration of symptoms reported a significantly lower level of sports activity (HSAS, 0-4; *P* < .009).

**Conclusion:**

Arthroscopic treatment for FAIS in elite athletes results in a statistically significant and clinically relevant improvement regarding symptoms, hip function, quality of life, and pain 5 years after surgery. Approximately half of the cohort was still in competitive sports at follow-up, yet 77% had decreased their level of sports. Nine of 10 patients were satisfied with their surgery.

Femoroacetabular impingement syndrome (FAIS) is a well-recognized cause of hip pain in the athletic population.^22,25^ In FAIS, an abnormal osseous morphology of the femoral head (cam) or acetabulum (pincer) generates pathological contact in the hip joint and can damage the labrum and cartilage. FAIS morphologies are observed in athletes presenting with hip pain, reduced range of motion (ROM) of the hip, and restrictions in sports participation.^40^ Athletes with high demands in terms of hip flexion, such as ice hockey and soccer players, run an increased risk of developing hip pain caused by cam and/or pincer deformities.^1,40^

Arthroscopic treatment for FAIS has become increasingly common during the past decade and has yielded good clinical outcomes.^16,17,31^ The purpose of surgical intervention is to reduce pain and increase ROM by removing cam and pincer deformities, striving for impingement-free hip anatomy and improved hip function. The literature also suggests a low rate of complications and reoperations after arthroscopic treatment for FAIS.^29^ Although several studies have reported positive results in elite athletes treated with arthroscopic surgery, most use return to sports (RTS) or performance outcome.^3,6,38^ However, only a few studies have used patient-reported outcome measures (PROMs) to present the results,^10,36,37,39^ and the reports on the long- or midterm outcome in this patient category are limited.^9,33^ Nevertheless, arthroscopic treatment in athletes presenting with FAIS is increasing, and studies suggest that high-level athletes will improve their hip function and have a high rate of return to preinjury activity levels.^2,4,7,8^

Follow-up after hip surgery generally includes PROMs. Early PROMs, such as the modified Harris Hip Score,^16^ were developed for an elderly population with osteoarthritis and are therefore not suitable for younger, more active patients.^21^ Lately, PROMs validated for young and active patients have been established; they include the International Hip Outcome Tool short version (iHOT-12)^15^ and the Copenhagen Hip and Groin Outcome Score (HAGOS).^43^ These scores are recommended by the scientific community for evaluating FAIS in this patient population.^14^

The aim of the present study was to evaluate the 5-year results after arthroscopic treatment for FAIS in elite athletes with PROMs validated for an active population. The primary hypothesis was that elite athletes experience improved outcomes 5 years after surgical treatment.

## Methods

Consecutive elite athletes undergoing arthroscopic treatment for FAIS between November 2011 and December 2012 were followed prospectively for 5 years. Patients were treated at 2 hospitals by 3 high-volume surgeons (M.S.). The outcomes at the 1-year follow-up were reported by Sansone et al^37^ for this particular cohort of patients.

The inclusion criteria were a diagnosis of FAIS, having undergone arthroscopic treatment after a nonsatisfactory result following nonsurgical treatment, and an age <40 years. To define “elite athlete,” the Hip Sports Activity Scale (HSAS) was used. The patients included in this study had an activity level of 7 or 8 before the onset of symptoms.^30^ An HSAS activity level of 7 corresponds to defined competitive sports with moderate physical demand at the elite level, such as alpine skiing, skating, and dancing, or more physically demanding sports in semielite leagues or at the collegiate level, such as soccer, ice hockey, tennis, martial arts, and track and field.

HSAS activity level 8 corresponds to more physically demanding sports at the elite level, such as soccer, martial arts, tennis, and track and field.^30^ A combination of symptoms, physical examination, and radiological findings formed the basis of diagnosis of FAIS in this study. Radiological findings corresponding to FAIS include cam, pincer, or both. The exclusion criterion was any surgical treatment on the studied hip before the arthroscopic treatment or a conversion to total hip arthroplasty within the 5-year follow-up period.

Preoperatively and 5 years after surgery, all the athletes were asked to complete a web-based questionnaire on the following PROMs:

- HAGOS, with 6 dimensions covering symptoms, pain, function in daily living, function in sports and recreation, participation in physical activities, and hip- and/or groin-related quality of life^43^
- EQ-5D and EQ-VAS (European Quality of Life–5 Dimensions Questionnaire and European Quality of Life–Visual Analog Scale)^34^
- A standardized instrument evaluating health-related quality of life^34^
- iHOT-12, a shorter version of the iHOT-33, developed to measure health-related quality of life and changes after the treatment of hip disorders in young and active patients^15^
- HSAS to measure the level of physical activity^30^
- A visual analog scale for hip function
- A single question regarding patient satisfaction (yes/no)

Apart from the single question regarding satisfaction, these PROMs have been validated and translated into Swedish.^20,32,42^ There was an unbiased evaluation of outcomes in terms of the analysis. Chondral damage was classified intraoperatively according to Konan et al.^24^

The study was approved by the regional ethical review board in Gothenburg (approval 071-12).

### Surgical Technique

All patients underwent individualized hip arthroscopy based on their history, radiology, clinical, and intraoperative findings.

An anterolateral portal and a midanterior portal were used with the patient in the supine position on a traction table. To examine the central compartment as the first step of the surgery, axial traction was applied. A ligament-sparing capsulotomy was used to reach the peripheral compartment. This longitudinal cleavage of the capsular ligaments could reduce the surgical trauma and the risk of iatrogenic hip instability. The surgical approach did not include capsular closure, since no transverse capsulotomy was performed. Pincer deformities were resected with an “over the top” technique, with the bur placed in the perilabral sulcus. In the event of small pincer resections, the labrum may be left in situ. However, with larger deformities leading to a clear separation between the labrum and the acetabular edge, the labrum was reattached with suture anchors.

Since determining the precise extent of any impingement deformity is complicated to determine, all femoral abnormalities were carefully resected. To accomplish this, the resection occurred at all the accessible parts of the femoral neck. Resection of eventual pistol-grip deformities was reached far posterior to the lateral retinacular fold, with careful attention paid to avoid damage to the lateral retinacular vessels, with possible osteonecrosis of the femoral head as a result. Perioperative fluoroscopy was continually used to control the extent of the bony resection.

Patients were permitted free ROM directly after the surgery, with weightbearing allowed, although crutches were recommended for outdoor walking the first 4 weeks after surgery. Physiotherapy was initiated immediately after surgery, and the intensity of the rehabilitation was progressively amplified in accordance with the patient and symptoms. The protocol comprised exercises for strength, endurance, stability, coordination, and ROM. To avoid heterotopic ossification, the patients were prescribed nonsteroidal anti-inflammatory drugs for the first month after the surgery. The surgical technique was previously described by Sansone et al.^37^

### Statistical Analysis

The data analyses were performed with SAS for Windows (v 9.1; SAS Institute). Descriptive statistics were used for demographic variables and presented as the mean, median, standard deviation, and range. Nominal data were reported as numbers and percentages. For comparisons of PROMs preoperatively and at the 5-year follow-up, the Wilcoxon signed rank test was used for continuous variables and the sign test was used for categorical variables. Correlations between the HSAS and continuous variables were calculated with Spearman correlation. A power analysis was conducted and enrollment undertaken accordingly. Significance was set at *P* < .05.

To evaluate the outcome with regard to the minimally important change (MIC), the number of patients exceeding the MIC was reported. MIC values were previously established for the iHOT-12 as 9.0 and for the HAGOS subscales as follows: 9.3, symptoms; 9.7, pain; 11.8, function in daily living; 10.8, sports; 13.1, physical activity; and 8.8, quality of life.^20,42^

## Results

In total, 79 consecutive elite athletes were eligible for inclusion and treated with arthroscopic surgery for FAIS. However, 18% did not participate in the 5-year follow-up. One of the patients had undergone a total hip arthroplasty at the follow-up and was excluded from the study. A total of 64 patients were therefore included in the final analysis: 52 (81%) men and 12 (19%) women with a mean ± SD age of 24 ± 6 years at the time of surgery. Twenty patients underwent bilateral surgery, generating a total of 84 operated hips. Six patients (9%) had undergone a reoperation during the follow-up period. The mean duration of symptoms before surgery was 31 months (Table 1).

**Table 1:** Patient Demographics^*a*^ (N = 64)

Bilateral FAIS	20
Age at time of surgery, y	24 ± 6
Male:female	52:12 (81:19)
Duration of symptoms, mo	31.0 ± 32.7
Body mass index	23.7 ± 2.1

a Values are presented as n (%) or mean ± SD. FAIS, femoroacetabular impingement syndrome.

Isolated cam morphology was seen in 43 athletes (51%); none had isolated pincer morphology; and 41 (49%) had a combination of cam and pincer morphology. The labrum was sutured in 8 hips (9%) and resected in 1 hip (1.2%) (Table 2).

**Table 2:** Performed Arthroscopic Procedures (N = 64 Patients and 84 Hips)

Diagnosis and Procedure	Hips, n (%)
Cam	43 (51)
Pincer	00 (0)
Combined (cam and pincer)	41 (49)
Labral repair	8 (9)
Labral resection	1 (1.2)
Microfracture	4 (4.8)

Chondral damage was found in 42 of 84 (50%) reported hips (Table 3).

**Table 3:** Cartilage Damage Classification According to Konan et al^24^ (N = 84 Hips)

	Hips, No.
0	36
1a	5
1b	0
1c	0
2	20
3a	14
3b	2
3c	0
4a	0
4b	1
4c	0
Not visualized	6

The most commonly performed sport was soccer, followed by ice hockey (Table 4).

**Table 4:** Type of Sport Before Symptoms

Sports Activity	Patients, No.
Soccer	39
Ice hockey	9
Handball	3
Dancing	2
Figure skating	2
Martial arts	2
Bandy	1
Badminton	1
American football	1
Basketball	1
Scooter-cross	1
Biathlon	1
Track and field	1

Preoperative scores as compared with those obtained at the 5-year follow-up revealed a significant improvement for the HAGOS subscales, EQ-5D, EQ-VAS, iHOT-12, and visual analog scale. At the 5-year follow-up, 90.5% of athletes reported satisfaction with their overall hip function (Table 5).

**Table 5:** Changes in Patient-Reported Outcome Scores From Presurgery to 5-Year Follow-up^*a*^

	Presurgery (N = 64)	5-y Follow-up (N = 64)	Change	*P* Value
HAGOS				
Symptoms	51.7 ± 19.9	71.9 ± 21.9	20.3 ± 22.2	<.0001
Pain	61.0 ± 18.5	81.1 ± 20.8	20.1 ± 20.3	<.0001
Function of daily living	67.1 ± 22.4	83.6 ± 21.8	16.5 ± 23.3	<.0001
Function in sports and recreation	40.0 ± 21.8	71.5 ± 27.0	31.5 ± 30.2	<.0001
Participation in physical activities	25.0 ± 25.7	67.4 ± 32.2	42.4 ± 39.0	<.0001
Hip- and groin-related quality of life	34.4 ± 21.4	68.0 ± 28.0	33.6 ± 27.5	<.0001
EQ-5D	0.60 ± 0.276	0.83 ± 0.220	0.23 ± 0.235	<.0001
EQ-VAS	66.1 ± 18.6	76.7 ± 18.1	10.6 ± 22.0	.0002
iHOT-12	40.0 ± 18.5	68.8 ± 29.3	28.8 ± 27.0	<.0001
VAS: hip function	49.2 ± 20.2	74.4 ± 21.0	25.6 ± 24.5	<.0001
Satisfaction with surgery				
Satisfied		57 (90.5)		
Not satisfied		6 (9.5)		

a Values are presented as n (%) or mean ± SD. EQ-5D, European Quality of Life–5 Dimensions Questionnaire; EQ-VAS, European Quality of Life–Visual Analog Scale; HAGOS, Copenhagen Hip and Groin Outcome Score; iHOT-12, International Hip Outcome Tool (short version); VAS, visual analog scale.

Improvement in the HAGOS subscales exceeded the MIC value for 73% of the patients in terms of symptoms, 69% for pain, 55% for activities of daily living, 73% for sports, 75% for physical activity, and 86% for quality of life. The improvement on the iHOT-12 exceeded the MIC value in 73% of all patients.

Of 64 elite athletes, 54% were still participating in competitive sports (HSAS, 5-8) 5 years after surgery. A decrease in level on the HSAS was reported by 77% of the patients (Table 6).

**Table 6:** Change in Hip Sports Activity Scale From the Period Before Symptoms to 5-Year Follow-up^*a*^

Level	Before Symptoms (N = 64)	5-y Follow-up (N = 64)
0		1.6
1		7.9
2		7.9
3		7.9
4		20.6
5		22.2
6		4.8
7	57	15.9
8	43	11.2

a Values are presented as percentages. Summary overall: decrease, 77.4%; equal, 21.0%; increase, 1.6%.

**Figure 1.**
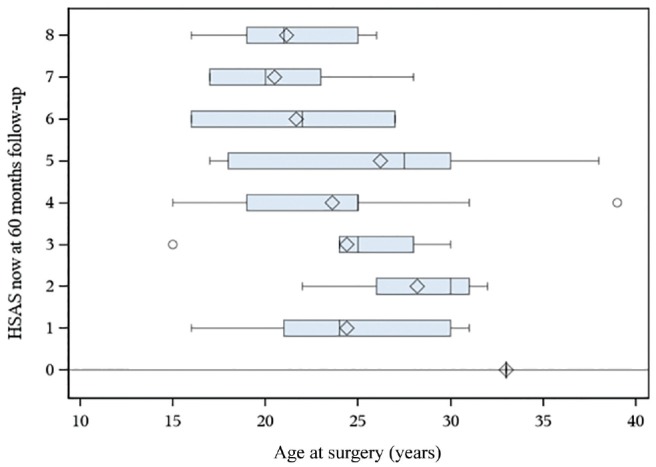
A box plot comparing the Hip Sports Activity Scale (HSAS) with age at surgery. The box is displaying the spread of the data where the quadrant is the mean value and the line next to it shows the median value. The whiskers display the minimum and maximum quartile value, and the dot indicates whether there is an outlier.

**Figure 2.**
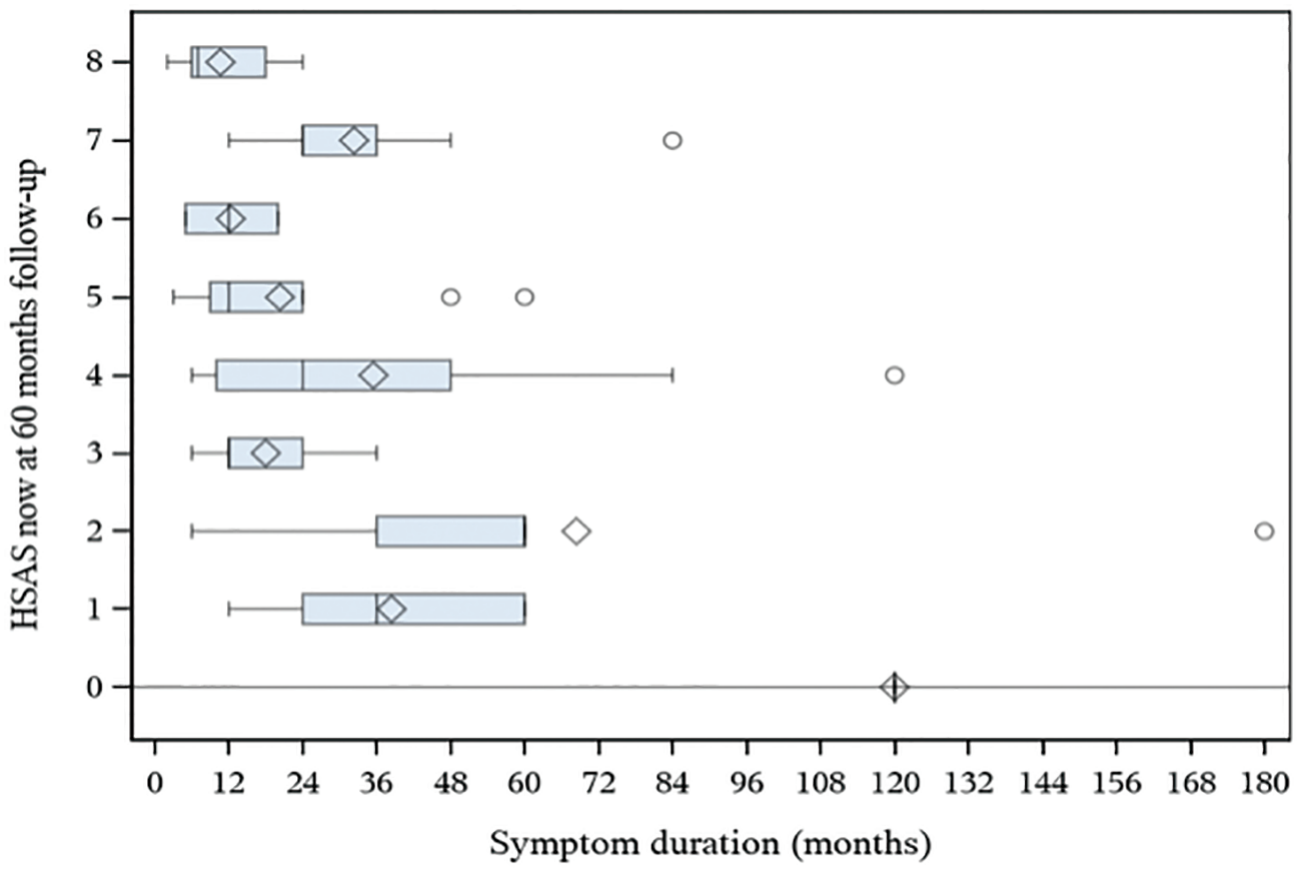
A box plot comparing the Hip Sports Activity Scale (HSAS) with symptom duration. The box is displaying the spread of the data where the quadrant is the mean value and the line next to it shows the median value. The whiskers display the minimum and maximum quartile value, and the dot indicates whether there is an outlier.

Lower levels of sports activity (HSAS, 0-4) were reported by patients with a higher age at the time of surgery, with a mean age of 30 years. Furthermore, lower levels on the HSAS were seen in patients reporting a longer duration of symptoms before surgery, at a mean of 41 months as compared with 21 months in patients with higher levels of sports activity (HSAS, 5-8; *P* = .013). These data were calculated with a correlation analysis and are shown as box plots in Figures 1 and 2.

## Discussion

The main finding in this study was the improvement in hip function 5 years after arthroscopic treatment for FAIS in elite athletes. Patient-reported hip function was improved in all PROMs, except for the HSAS, from presurgery to the 5-year follow-up. Nine of 10 patients reported satisfaction with their overall hip function. The average improvements exceed the MIC for the iHOT-12 and HAGOS, indicating a clinically relevant improvement.

In accordance with the present study, several studies have reported favorable outcomes after the arthroscopic treatment of FAIS in athletes.^9,11^ However, these studies used older PROMs not validated for an active population. The PROMs included in the current study have been validated for an active population and are therefore suitable for the cohort of elite athletes. Moreover, the HAGOS and iHOT-12 are recommended for use in assessing treatment for FAIS.^14^

Perets et al^33^ reported the 5-year outcome in athletes, including the iHOT-12 with a mean value of 78.8 at follow-up. Sansone et al^37^ published the 1-year follow-up outcomes for the present cohort with outcomes similar to those in this current study, suggesting an average constancy in outcome and hip function over time.

Despite remaining in sports, many athletes reported a reduction in the level of sports in which they participated 5 years after surgery. Interestingly, patients still reported an overall satisfaction with their hip function. There may be several explanations for this. Most patients who reported a decrease in the HSAS were older, and it is not known how long they would have remained in competitive sports regardless of hip pain and FAIS. The level of the HSAS is expected to decrease after 5 years, independent of the function of the hip, given that the probability of retirement from sports increases with age. Moreover, a decrease in the HSAS was reported in patients with a longer duration of symptoms before surgery (mean 41 months); thus, elite athletes with a long symptom duration may have difficulty returning to a high level of sports. Menge et al^28^ followed the career lengths of hockey players after arthroscopic treatment for FAIS and reported a correlation between a longer career and younger age, as well as shorter symptom duration at the time of arthroscopy. It is also possible that longer symptom duration is related to a higher degree of cartilage damage, which could affect the load capacity of the joint and thereby the ability to participate at elite levels.

A systematic review by Reiman et al^35^ reported a 74% rate of RTS after arthroscopic treatment for FAIS, and other studies have cited rates up to 90% RTS.^44^ However, most studies do not use clear definitions of RTS, and sports performance is seldom measured. RTS rates are different depending on the type of sports, and they differ among studies.^19,27^ Ishøi et al^18^ assessed RTS with a strict definition and reported a lower rate than previously described in the literature, 57% overall; however, only 17% of athletes returned with optimal sports performance and participation.

The HSAS, as included in this study, is a validated outcome with a clear definition in terms of the level of sports, making it easy to compare preinjury and postoperative levels of sports activity and enabling the inclusion of patients from several sports. Of the patients in this study, 54% reported participating in competitive sports (HSAS, 5-8) 5 years after the surgical procedure, but only 21% were on the same level of sport. Previous data on the same cohort indicated that 73% of the patients still participated in competitive sports 1 year after surgery.^37^ However, it is not surprising that 46% did not remain in competitive sports after 5 years, as the mean age at the time of surgery was 24 ± 6 years. This could be due to a natural attrition rate (ie, decrease) in level of sports. Future studies should use a strict definition of RTS to report a more exact rate.

FAIS is common among athletes in sports with high demands on the hip, such as soccer and ice hockey.^1,7,26^ The patients in the current study were mostly soccer and ice hockey players. However, other studies have reported good patient-reported outcomes after arthroscopic treatment for FAIS in other sports involving repetitive flexion and rotational loading on the hips, such as cycling and yoga,^12,13^ further indicating arthroscopic surgery as a viable treatment option for patients with FAIS who are performing sports other than soccer and ice hockey.

Labral repairs were performed in only 9% of the patients in this study. This is due to the technique used and the pathophysiology. This rate is lower than that reported in many other publications. For example, Perets et al^33^ performed labral repairs in 75.8% of the surgical procedures. The discrepancy between this study and others might be due to the method by which pathophysiology was evaluated and the disparities in treatment customs among countries. The importance of chondrolabral pathology is not yet fully understood.

Strengths of this study are the long follow-up period, the prospective data collection, the updated PROMs validated for a young and active population, and a well-defined cohort of elite athletes. According to the methodological index for non-randomized studies (MINORS), this study receives 13 of 16 possible points, indicating high quality.^23,41^

Limitations of this study include the absence of a control group. Of the patients meeting the inclusion criteria, 82% participated in the follow-up. The remaining 14 patients (18%) were lost to follow-up, and this could bias the results. Moreover, the study did not include objective radiographic values, such as magnetic resonance imaging or radiographs with alpha angle^5^ for cam morphology, a value frequently used in describing and diagnosing FAIS, which reduces the opportunity to compare the present results with those in other studies. Nevertheless, the alpha angle is debated as a single predictor of FAIS, and the diagnosis in this study was based on radiographic findings, symptoms, and clinical findings, as suggested by the Warwick agreement.^14^ Another limitation is the long duration of symptoms and relatively high age of some patients, which could bias the RTS rate. The study was not blinded; however, the data analysis was performed in a blind manner.

## Conclusion

Arthroscopic treatment for FAIS in elite athletes results in a statistically significant and clinically relevant improvement regarding symptoms, hip function, quality of life, and pain 5 years after surgery. Approximately half of the cohort was still in competitive sports at follow-up, yet 77% had decreased their level of sports. Nine of 10 patients were satisfied with their surgery.
